# Pharmacovigilance and Pharmacoepidemiology

**Published:** 2008-10

**078**

**Profile of voluntarily reported non-cutaneous adverse drug reactions: A six month prospective study in a government hospital**

Surendra SA^1^, Dang A^2^, Rataboli PV^3^

**^1^Goa Medical College, Goa, India; ^2^Goa Medical College, Goa, India; ^3^Goa Medical College, Goa, India.**

**Introduction:**

Major chunk of India’s population prefers government hospitals to seek health care facilities. Voluntary reporting of Adverse Drug Reactions (ADRs), if any, mainly reports cutaneous drug reactions in such institutions, as it is easily assessed subjectively.

**Aim**

To analyze the profile of voluntary reported non-cutaneous ADRs in a government hospital.

**Methods:**

Pharmacovigilance set up was made available in all clinical departments and physicians were encouraged to notify suspected ADRs. All the notified ADRs were collected and analyzed.

**Results:**

After 6 months of ADR monitoring 74 non-cutaneous ADRs were reported. Maximum ADRs were reported in the age group of 20-30 years (24%). Males showed more ADRs (65%) than females (35%). Both oral and parenteral routes of administration were responsible equally in the ADR causation. CNS involvement was seen most commonly (21%) followed by CVS (17%) and GIT (14%). Among the drugs, ß-lactam antibiotics were implicated maximum number of times (17) followed by fluoroquinolones (10) and various cytotoxic drugs (9). The number of “Type I” and “Type II” ADRs reported, as per Rawlins and Thompson’s classification, were almost equal. Causality asse	ssment was done by Naranjo Algorithm and 51 ADRs were seen to fall in “probable category” as compared to 17 in the “highly probable” one. Out of the 74 non-cutaneous ADRs reported, 17 were “severe” in accordance with Modified Hartwig and Siegel’s scale, out of which 6 patients died.

**Conclusions:**

Voluntary reporting system of ADRs has proved to be successful in reporting a variety of non-cutaneous ADRs in our hospital.

**079**

**A cyber survey on awareness on emergency contraception among Indian population**

Sreekanth G^1^, Shivali R^1,^ Parvathavarthini S^1^, Usha Rani V^2^

**^1^Sri Ramachandra Medical College, Chennai, India; ^2^S.V. Medical College, Tirupathi, India.**

**Background:**

We conducted an internet survey to assess “awareness on Emergency Contraception” and at the same time spreading the awareness among respondents in the context of increased usage and availability of Emergency Contraceptive Pills (ECPs) as OTC drugs.

**Method:**

A survey questionnaire was prepared based on similar studies. Response was sought using emails and internet blogs. Measures were taken to protect the anonymity of the respondents. Data was sorted out based on the country of the respondent and analysed. After the survey, respondents were given proper information on ECPs; there by spreading awareness same time they answer the survey. Survey is still ongoing.

**Results:**

Within 2 months of launching the survey, there were a total of 92 respondents (81 Indians). Respondents were categorized into groups based on age group, occupation and living status. 83.9% replied that they were aware about emergency contraception. Only 58.1% said that ECPs has to be taken within 72 hours of intercourse. Menstrual irregularities are the most commonly aware side effect of ECPs followed by lower abdominal pain (56.8%, 29.6% respectively). 75.3% knew that ECPs were available as OTC drugs. Only 54.3% knew all the situations where ECPs can be used. People were not clear about the actual mechanism of action of ECPs and only 24.6% knew this. Around 13% people felt ECPs can be abortion, regular contraception pills.

**Conclusion:**

Survey revealed mixed results on awareness. But providing answers to the questions could increase the awareness which is done by a post survey internet page on proper information.

**080**

**A retrospective study of the drug utilization pattern and adverse drug reactions in hiv positive cases receiving anti-retro viral therapy in art center, Gauhati Medical College and Hospital**

Dey S, Deb T, Lahkar M

**Gauhati Medical College, Guwahati, India.**

**Objectives: 1.**

To study the drug utilization pattern of different regimens of ART in HIV positive cases in GMCH. 2. To study the pattern of different adverse drug reactions in the ART regimens. 3. To study the pattern of occurrence of opportunistic infections in HIV positive cases.

**Method:**

The study was conducted among HIV positive cases registered in the ART center of Gauhati Medical college(GMCH) from 12/06/06 to 11/12/07.The data was derived retrospectively from records maintained by the ART Centre as ‘White Card’ for every patient and was collected in a pre-designed proforma.

**Results:**

The commonest regimen used as a ‘start on‘therapy was d4T30/3TC/NVP accounting for 76 out of 161 cases i.e., 47.20% cases. The common adverse drug reactions in descending order of frequency were gastrointestinal(60.25%), neurological(20.49%), liver dysfunction (14.90%), lipid function dysfunction(11.80%), anemia(9.94%), dermatological (7.45%), autonomic(5.59%), lipodystrophy(4.35%), palpitation(1.87%), renal colic (1.24%), cramping pain (1.24%), Thyroid dysfunction(1.24%). The commonest opportunistic infection found in these 161 cases was Tuberculosis, 78 cases (43.48%) with Pulmonary TB(32.92%) and Extrapulmonary TB(12.42%).

**Conclusion:**

In this study d4T30/3TC/NVP was found to be the most common ‘start-on’ regimen in HIV positive cases in Anti-retroviral therapy centre of GMCH. Among 161 cases studied 60.25% cases presented with gastrointestinal disturbances clearly being the most common adverse effect. Tuberculosis was found to be the commonest opportunistic infection in these patients

**081**

**Prescription pattern of antiepileptic drugs in epilepsy clinic patients in a tertiary care hospital**

Tandon S, Kaushal S, Chopra SC, Singh G

**Dayanand Medical College and Hospital, Ludhiana, India.**

Epilepsy requires long term treatment in which monotherapy is preferred and polytherapy used only if there is inadequate control of seizures. The choice of medication depends on several factors. This study was undertaken to study the prescription pattern of AEDs in the first three years of epilepsy clinic at DMCH. The data of all the 558 epilepsy clinic patients was studied for demographics, semiology, etiological diagnosis, seizure record, drugs and doses used. This data is being reported for 326 patients (1-90 years of age; 204 males and 122 females) followed up for 875 patient years (till seizure cessation). This excludes 108 patients with non-epileptic seizures and 124 patients lost to follow up after few visits. In first year of treatment, monotherapy was instituted in 77.35% patients; 2 drugs in 14.4%; 3 drugs in 4.6% and more than 3 drugs in 0.9%; 2.8% did not receive any AED in 1^st^ year but had to be prescribed AEDs subsequently. Amongst monotherapy, carbamazepine (32.5%), valproate (29.4%) and phenytoin (25%) were used in first year. Monotherapy was used for 672 patient years. Most common add on drugs were clobazam (32.4%) and clonazepam (9.4%). At the end of three years, out of 326 patients, 29.1% (95) had to be continued on AEDs due to inadequate control while 70.8% (231) could be successfully tapered off the medication. Monotherapy is being preferred at the epilepsy clinic. Carbamazepine is the most commonly prescribed AED for monotherapy and clobazam as add-on.

**082**

**A prospective analysis of serious adverse drug reactions reported in patients at Civil Hospital Ahmedabad, a tertiary care teaching hospital**

Mor MN, Gandhi AM, Desai CK, Desai MK, Dikshit RK

**B.J. Medical College and Civil Hospital, Ahmedabad 380016, India.**

**Introduction:**

Serious adverse drug reactions (ADRs) are the 4th to 6th leading cause of death in USA. Present study was carried out to evaluate the pattern, causality and preventability of serious ADRs in a tertiary care teaching hospital.

**Materials and Methods:**

This prospective observational study was carried out from June 2005 to May 2008 at different departments of Civil Hospital, Ahmedabad. Adverse Drug Event Reporting Form (CDSCO) was used to record the ADRs. Serious ADRs as defined by the WHO criteria were further analysed. Causality assessment was carried out using the WHO-UMC criteria and Naranjo’s algorithm and Preventability was assessed using modified method of Schumock and Thorton (1991).

**Results:**

Out of 1139 cases reported during the period, 282 (24.75%) were serious ADRs. Males were more frequently affected (Male: Female = 1.5:1). Most common age group affected was 21 – 40 years. Commonly observed clinical presentations were Stevens Johnson Syndrome (18.53%) and Cutaneous ADEs (17%). Most common causal groups were antimicrobials (18%), antitubercular drugs (18%) and antiretroviral drugs (15%). Among the serious ADRs, 61.24% patients required hospitalization. Death occurred in 4.26% cases. Causality analysis of the reports showed that most drugs were “Possible” or “Probable” causal agents. Out of 258 cases, 34 (13.17%) ADRs were definitely or probably preventable.

**Conclusions:**

Serious ADRs account for hospital admissions, increased morbidity, prolonged treatment and in some cases death, as evidenced by our study. Hence ADR monitoring is one of the measures that can help reduce the burden of these ADRs and improve the benefit versus harm ratio of drugs.

**083**

**A retrospective study of the drug prescribing pattern in acute myocardial infarction**

Kamath A, Shanbhag T, Shenoy S, S Ramesh

**Kasturba medical college, Manipal, India.**

**Introduction:**

There is still a noteworthy underuse of reperfusion in acute myocardial infarction, as well as a deficit in the prescription of statins, antiplatelet drugs, ACE inhibitors or beta blockers along with gender and age based differences. The aim of our study was to determine the prescribing rate of drugs on hospital admission and at discharge in patients with acute MI with regard to age and gender.

**Methods:**

A retrospective study of the case files of 349 patients admitted to our hospital with a diagnosis of acute MI during the year 2004-2006 was done. The age, gender, drugs prescribed during the 3 days following admission and on discharge, presence of co-morbidities, outcome and duration of stay was noted for each patient.

**Results:**

Of the 349 patients, 81% were males and 19% females. 40% were more than 65 years of age. The year-wise prescription rate in males showed a consistent increase except for thrombolytics. The prescribing rate on admission was lower in females during the year 2004 which became comparable to males in the year 2006. Prescription of drugs on admission was lower in elderly but was comparable on discharge. The average duration of hospitalization was 8.05 days.

**Conclusion:**

The prescription rate of thrombolytics, beta blockers, ACE inhibitors was low on admission but higher on discharge. This may be due to initiation of treatment following stabilization of the cardiovascular status of the patient. The prescription rate in females showed a consistent increase with each year reflecting effective implementation of treatment guidelines.

**084**

**Epidemiology of hypertension among diabetes patients**

Patel DM^1^, Patel Rameshvar K^1^, Kanzariya NR^1^, Patel MP^1^,

Patel MM^2^, Patel NJ^1^

**^1^S.K. Patel College of Pharmaceutical Education And Research, Kherva ^2^Kalol Institute of Pharmacy, Kalol, India.**

**Objective:**

The purpose of this study was to examine the prevalence of hypertension among diabetic patients associated with cardiovascular dysfunctions (CVDs) in North Gujarat region.

**Methods:**

Diabetic patients were surveyed at different hospitals of North Gujarat area. As per the questionnaires patients were included and excluded from the study. Finally statistical analysis of data was performed.

**Result:**

The study suggested the influence of genetic components in the development of hypertension among diabetic patients, but further studies are required for confirmation. Duration of diabetes was found major factor for the development of hypertension among diabetic patients. There was higher prevalence of metabolic syndrome and hypertension among people with obesity. Sedentary life style and stress were not found as risk factor for the development of hypertension among diabetic patients with CVDs. Blood glucose and blood pressure level were significantly restored after drug treatment as compared to initial levels. In drug treatment, combination of sulfonylurea and biguanides was found as the most prescribed drug as compared to other anti-diabetic drugs while for the treatment of high blood pressure in diabetic patients ACE inhibitors were found most prescribed anti-hypertensive drug. Surprisingly people were found to be aware of beneficial effect of meditation/yoga.

**Conclusion:**

Hypertension is the most prevalent disease associated with diabetes, so management of blood pressure is also essential with blood glucose management in diabetic patients.

**085**

**Audit of prescribing pattern and drug use in medicine department of tertiary care hospital**

Pimpale S^1^, Jadhav P^2^, Gore R^3^, Moghe V

**MGM Health University, Kamothe, Navi Mumbai, India.**

**Introduction:**

Medical audit is concerned with the observance of standards of medical treatment at all levels of the healthcare delivery system^1^. Drug utilisation studies are a part of medical audit and seek to monitor, evaluate and modify, if necessary, the prescribing habits of practitioners. The goal is to make medical care more rational and cost effective^1^. It is necessary to regularly evaluate the prescribing trends of practitioners to identify patterns of drug use and subject it to standard indicators like WHO core drug use indicators to gather information about changing trends in prescriptions as well as disease patterns.

**Methods:**

All the prescription cards from the medical OPD of the MGM teaching hospital over a week (01.06.08 to 07.06.08) were taken for analysis. The number of drugs prescribed in each prescription was taken into account to calculate: 1) the total number of drugs prescribed, 2) the incidence of polypharmacy, 3) percentage of drugs prescribed by brand/generic name. The data from the records were entered into a specially designed proforma. The following parameters were recorded for each prescription: patient’s OPD number, age, sex, diagnosis (patient information); drug name, dose, frequency and duration of prescription (drug information). The above data was subjected to WHO drug use indicators to draw conclusions.

**Results:**

Out of total 460 drugs from 100 prescriptions, 76% were prescribed by brand name. Antibiotics accounted for 52.17% which majorly included newer class.

**Conclusions:**

There was high incidence of polypharmacy and prescribing by brand name which increases overall cost of prescription.

**086**

**Self medication pattern among medical students in M.L.N. Medical college, Allahabad**

Shukla AK, M Anand, Chugh Y, Sharma A, Yadav VS, Chaurasia RC, Kastury N

**MLN Medical College, Allahabad, India.**

**Objective:**

To study the self medication pattern among medical students of M.L.N. medical college Allahabad.

**Materials and Methods:**

The study was conducted by using interview method based on pre designed questionnaire. Respondents were randomly chosen students (n=185) of 1^st^, 2^nd^, final professional and interns. Questions were asked regarding use of medications in past one year and its indications.

**Result:**

82% of the students admitted taking medications frequently without prescriptions while the rest did so occasionally. Most of the students (95%) took NSAIDs. In other categories the use was found to be antihistaminics (60%), antibiotics (50%), nutritional supplements (48%), antitussives (45%), proton pump inhibitors (35%) and antacids(30% of the students). About 10% of the students frequently used antianxiety or sedative drugs and in this category 75% students were of final professional or interns. The commonest indication for self medication was headache followed by fever. All the users checked correct name, intactness of package and expiry date of the drugs before use. 90% of them were aware of the possibility of adverse drug reactions.

**Conclusion:**

A large number of medical students are involved in self medication practice. Use of anti anxiety and sedative drugs was high particularly among final professional students and interns possibly due to day by day increasing pressure of PGME examinations.

**087**

**Risk benefit evidence of clopiodgrel vs aspirin in prophyalxis of cardiovascular disease in patients of chronic kidney disease**

Dash A, Raghavendra M, Pandey BL, Ojha JP, Singh A

**Institute of Medical Sciences, Banaras Hindu University, Varanasi-221005, India.**

**Objective:**

To assess the efficacy, safety and cost effectiveness of clopidogrel over aspirin with regard to renal function, platelet aggregation and C-Reactive protein (as an inflammatory marker) in chronic kidney disease patients.

**Methods:**

The study was carried out in patients of chronic kidney disease with serum creatinine level >1.8mg/dl with risk of cardiovascular disease; but no history of bleeding disorders, peptic ulcer disease, exposure to non-steroidal anti-inflammatory drug for last 1 month and not on dialysis. There were two treatment groups-Aspirin and Clopidogrel, (n = 35), in whom 75 mg aspirin and 75 mg clopidogrel was given respectively as an add-on therapy to the concurrent medications daily for 30 days. Blood samples were collected, on 0th day and on 31st day of treatment for assessment of renal function parameter, platelet aggregation (induced by ADP) and quantitative estimation of C-Reactive Protein. The overall cost of treatment was noted during 1 month of therapy. Students t-test was used for the statistical analysis.

**Result:**

When compared to clopidogrel, Aspirin significantly decreased creatinine clearance (*P*<0.045); and, led to a significant rise in serum uric acid (*P*<0.023) and serum potassium levels (*P*<0.001). Aspirin did not confer any added advantage over clopidogrel in decreasing platelet aggregation (*P*>0.05) or C-Reactive Protein level (*P*>0.05).The cost of one month therapy with clopidogrel was marginally (4.75%) higher than that of aspirin during the same period.

**Conclusion:**

We conclude; superior platelet anti-aggregatory effect, better lowering of C-Reactive protein and safety on renal function of Clopidogrel over Aspirin and at an affordable cost makes clopidogrel an efficacious but safer alternative to aspirin in patients of Chronic Kidney Disease who require prophylaxis of Cardio Vascular Disease.

**088**

**Anti cancer effects of common Indian toad (*Bufo melanostictus*) egg extract on experimental models**

Dasgupta SC, Gomes A

**University of Calcutta, 92 A P C Road Kolkata 700 009, India.**

**Introduction:**

Common Indian Toad (*Bufo melanostictus*) is identified as particularly convenient and useful resources of various bioactive compounds having therapeutic potential. In the present study an attempt has bee made to explore the antiproliferative and apoptogenic effects of this toad egg extract on EAC cells bearing mice and human leukemic cell U937 and K562.

**Methods:**

Toad eggs was collected and methanolic extract was prepared (TEE).It was dissolved and expressed in terms of dry weight. Anticancer activity was monitored on EAC cell by assessing tumour cell growth, dose dependent assay and survival time. Human leukemia cell line U937 and K 562 were used to study cell proliferation inhibitory effects and cytotoxic activity was evaluated through MTT assay. Morphological changes due to TEE treated U937, K562 and EAC cells were examined through bright field and florescent microscopy studies.

**Result:**

TEE (100*µ*g/20gm × 10 days) produced significant inhibition of EAC cell count, decreased EAC viability and increased EAC survival time as compared to control group. TEE (10*µ*g/ *µ*l) produces significant inhibition of U937 and K562 cell count. It also produced significant inhibition of MTT assay of U937 and K 562 cells. Fluorescent and bright field microscopy studies with EAC, U937 and K562 cells revealed membrane blebbing accompanied with nuclear fragmentation and membrane disruption as compared to control cells.

**Conclusion:**

From the present studies it may be concluded that TEE has anticancer activity. Further work on the active compounds and mechanism of action are warranted in future.

**089**

**Epidemiology of coronary heart disease among diabetes patients**

Kaur R^1^, Patel Rameshvar K^1^, Kanzariya NR^1^, Patel MP^1^, Patel MM^2^, Patel NJ^1^

**^1^S.K. Patel College of Pharmaceutical Education And Research, kherva, India; ^2^Kalol Institute of Pharmacy, Kalol, India.**

**Objective:**

The purpose of this study was to examine the prevalence of hypertension among diabetic patients associated with cardiovascular dysfunctions (CVDs) in North Gujarat region.

**Methods:**

Diabetic patients with cardiovascular disorders were surveyed at different hospitals of North Gujarat area. As per the questionnaires patients were included and excluded from the study. Finally statistical analysis of data was performed.

**Results:**

The risk for diabetes patients with coronary heart diseases (CHD) appears to result from a combination of genetic predisposition (family history) and lifestyle changes. Duration of diabetes (mostly 2-10 years) was also shown to influence the coronary heart diseases among diabetic patients associated with diabetes. Moreover, more than 70% diabetic patients with cardiovascular dysfunction were found male. Obesity was not the representing factor for CHD among diabetic patients with cardiovascular dysfunctions (CVDs) rather the sedentary lifestyle was found to be one of the primary drivers affecting the genesis of CHD. The changes in dietary habits (saturated fat) were major contributor as evident from the higher prevalence of CHD among diabetes patients (mixed / non-vegetarian food habit). Review of the drugs prescribed to the diabetic patients showed that combination of sulphonylureas with biguanides found to be prescribed the most. Full vascular protective measures with anti-platelets and antihyperlipidemic drugs were commonly practiced drugs.

**Conclusions:**

Appropriate lifestyle intervention would greatly help in preventing or postponing the onset of lifethreatening complication - CHD associated with diabetes and thus reducing the burden on the community.

**090**

**Prospective analysis of adverse drug reactions in geriatric patients at Civil Hospital, Ahmedabad**

Doshi M, Shah SP, Solanki MN, Desai CK, Desai MK, Dikshit RK

**B.J. Medical College and Civil Hospital, Ahmedabad-380016, India.**

**Objective:**

To evaluate and assess the causality, severity and preventability of geriatric adverse drug reactions (ADRs) in a tertiary care teaching hospital.

**Materials and Methods:**

This Prospective observational study was carried out from June 2005 to May 2008 at different departments of Civil Hospital, Ahmedabad. Adverse Drug Event Form devised by CDSCO was used to record the observations. ADR was defined using WHO criteria. Causality assessment was carried out using WHO-UMC criteria and Naranjo’s algorithm. Severity was assessed by Hartwigs scale and preventability by the modified method of Schumock and Thornton 1991.

**Results:**

Out of total 1139 ADRs reported, 70 were found in geriatric (≥65 years old) patients. The drugs causing ADRs were antibacterial 19 (21%), calcium channel blocker 8 (9%), antiplatlet 8 (9%), angiotensinconverting enzyme inhibitors 8 (9%) and NSAIDs 7(8%). The most common ADRs were pertaining to gastrointestinal tract (GIT). Patients having more than one associated medical disorder were 38 (54%), hypertension being the most common. Out of 70 patients, 46 (66%) received more than three drugs. Most ADRs were of probable or possible grade. Majority of ADRs required the suspected drug to be discontinued and/or addition of another drug as per Hartwig scale. About 9% ADRs were avoidable.

**Conclusion:**

ADRs in geriatric population are common. The common manifestations of ADRs in geriatric patient are pertaining to GIT and antibacterials are the common causal drug. Factors such as polypharmacy and coexisting diseases may contribute to the incidence and severity of ADR in this population.

**091**

**Study of drug induced cutaneous manifestations at civil hospital Vadodara**

Shah MK, Hotchandani SC, Bhatt JD

**Medical College, Vadodara, Gujarat, India.**

This study has reported cases of adverse cutaneous drug reactions (ACDRs) both as outpatients and in patients in S.S.G hospital, Baroda, Gujarat from October 2005 to September 2006.

**Materials and Methods:**

Total 70 cases, who had certain, probable or possible relation according to WHO causality assessment criteria were included in the study. All ACDRs were assessed and analyzed for incidence, drug class, individual drug causing ADRs, type of cutaneous reactions and various predisposing factors. Diagnosis and treatment of ACDRs were done by dermatologists of the hospital.

**Results:**

Most of the patients of ACDRs were in the age group 11-50 years. Male patients had predominance over female patients. The commonest morphological types of ACDRs were fixed drug eruptions (37.1%) and maculopapular rash (28.6%). Stevens- Johnson syndrome, dapsone syndrome and exfoliative dermatitis were the serious ACDRs. Antimicrobials (61.4%), nonsteroidal anti-inflammatory drugs (NSAIDs) (22.9%) and antiepileptic drugs (10%) were the most prominent drug group responsible for ACDRs. Maximum numbers of ACDRs were due to cotrimoxazole followed by ibuprofen, paracetamol and phenytoin. ACDRs to some new drugs were also observed like nevirapine induced maculopapular rash, oral isotretinoin induced oral cheilitis and vitamin B complex induced maculopapular rash. ‘ADR alert cards’ were given and they were asked to produce the same while visiting the physician in future.

**Conclusion:**

The observations made in our study emphasize the need for a strict and efficient pharmacovigilance system which could curtails the incidences ACDRs in clinical practice.

**092**

**Profile of voluntarily reported cutaneous adverse drug reactions (ADRS) in a tertiary care hospital: A six month prospective study**

Dang A, Rataboli PV

**Goa Medical College, Goa, India.**

**Introduction:**

Voluntary reporting of Adverse Drug Reactions (ADRs) was never a part of medical practice in Goa Medical College, the only tertiary care hospital serving the state.

**Methods:**

“ADR notification drop boxes” were made available in all the clinical departments. Physicians were encouraged to notify suspected ADRs by means of “ADR notification forms”. Duly filled forms were supposed to be dropped in the box. All the notified ADRs were reported to the pharmacovigilance centre under National Pharmacovigilance Programme.

**Results:**

After 6 months of ADR monitoring, 174 ADRs were reported, out of which 100 belonged to “cutaneous” category. Maximum ADRs were reported in the age group of 30-40 years (33%). Females showed more ADRs (55%) than males (45%). Oral route was responsible in the ADR causation in 73% cases as compared to parenteral route (27%). “Maculopapular rash” was the commonest presentation (51%), others being “urticaria” (16%), “SJS and EM” (7% each). Among the drugs, anti-epileptics were implicated maximum number of times (22) followed by fluoroquinolones (16) and β-lactam antibiotics (16). Causality assessment was done by Naranjo Algorithm and 56 ADRs were seen to fall in “probable category” as compared to 38 in the “highly probable” one. 65% and 34% of ADRs belonged to “mild” and “moderate” category respectively in accordance with Modified Hartwig and Siegel’s scale. Outcome of ADRs revealed that 35 patients were hospitalized or had prolonged hospitalization. One death was reported.

**Conclusions:**

Voluntary reporting of ADRs has now picked up as a routine practice by physicians in our hospital.

**093**

**Adverse drug reaction profile of antiepileptic drugs in patients attending epilepsy clinic in a medical college hospital of North India**

Kaushal S, Tandon S, Chopra SC, Singh G

**Dayanand Medical College and Hospital, Ludhiana, India.**

Adverse drug reactions (ADRs) are noxious and unwanted events necessitating a change in dose, addition of another drug, advising precautions or even stoppage of therapy. Antiepileptic drugs (AEDs) are selected and used for durations ranging from a few months to lifelong therapy. Due to relatively longer duration of treatment the frequency of ADRs is high. The data generated in first three years of epilepsy clinic of DMCH is presented herewith. Data from all the 558 patients attending the epilepsy clinic during the first three years of its existence was studied for ADRs reported and interventions instituted. The ADRs reported were then clubbed into major groups and frequencies as per drugs used were determined. The AEDs causing these ADRs were identified on the basis of causality assessment. The most common ADRs included sleepiness (13.4%); headache (6.6%); shaky hands (2.15%); trouble with mouth and gums, blurred vision and vertigo (1.9% each); dizziness and upset stomach (1.6% each); weight gain and menstrual abnormalities (1.2% each); irritability (1.1%); memory, hair loss, aggression (0.9% each); others (3.2%) and miscellaneous ADRs (4.1%). Sleepiness was reported with carbamazepine (57.3%); phenytoin and valproic acid (32% each) and clobazam (30.7%). Headache was reported with carbamazepine (67.6%), phenytoin (32.4%) and valproic acid (18.9%). One patient on phenytoin reported pain and stiffness in hip joint which improved with reduction in dose.

**094**

**Adverse drug reaction monitoring in a tertiary care hospital of eastern India: An overview**

Mohapatra S, Mohanty M, Panda R, Samal V, Rath K, Pradhan D, Devi S

S. C. B. Medical College, Cuttack, Orissa, India

Adverse drug reaction (ADR) monitoring though an integral aspect of rational therapeutics is at its infancy at all levels of health care due to lack of spontaneous reporting culture amongst the prescribers. To bridge this gap in our Institution, this study was undertaken at the Peripheral Pharmacovigilance Centre, under the aegis of its Co-ordinator, to study the impact of pharmacovigilance sensitization programmes on the reporting practice and to create a local ADR database and evaluate it. This study was conducted over a period of two years. At the outset various sensitization programmes were conducted in various clinical disciplines of our Hospital, coordinators were selected at each these departments and ADR Reporting Forms and contact numbers were distributed. Intermittent sensitization schedules were also conducted. The ADR data was pooled an analysed. Causality assessment was done according to Naranjo’s scale and severity assessment by ICH guidelines. A total of 258 ADRs were reported over a span of two years. There was an unequal incidence of reporting among the different disciplines, with some disciplines totally resistant to the intermittent sensitization processes. Maximum number of ADRs were caused by anti-psychotics and anti-epileptics. Most of the ADRs were of mild nature and involved the skin, gastro-intestinal system and central nervous system. Causality assessment showed most of them to be of probable category. A few undocumented ADRs were unearthed. ADR Monitoring though a very sensitive tool for rational therapeutics, much more needs to be done for its effective implementation in order to reach this ultimate goal.

**095**

**A comparative study of causality assessment scales used in the analysis of spontaneously reported events: WHO-UMC criteria vs naranjo probability scale**

Sharma M^1^, Gupta SK^2, 3^, Gupta VB^1^, Chatterjee A^4^

**^1^B.R. Nahata College Of Pharmacy, Mandsaur, India; ^2^Dean And Director General, Institute Of Clinical Research India, New Delhi, India; ^3^Emeritus Professor, Delhi Institute of Pharmaceutical Sciences and Research, New Delhi, India; ^4^Panacea Biotec, Ltd, New Delhi, India.**

**Introduction:**

Spontaneous adverse drug reaction (ADR) reporting has been a major source of information in pharmacovigilance. The primary purpose of spontaneous ADR reporting is to provide early warnings or “signals” of previously unrecognized drug toxicity. The methods that have been developed for the estimation of the probability that a drug caused an adverse event(s) mainly include; the WHO-UMC criteria, the Naranjo probability scale, the Kramer scale and the Karch and Lasagna scale. None of the methods have shown to produce a defined and consistent likelihood of relationship.

**Objective:**

To quantitatively estimate and compare the relationship between the WHO-UMC causality assessment criteria and the Naranjo probability scale.

**Methods:**

A non interventional observational study was carried out in India to capture suspected adverse drug reactions (ADRs) caused by Extended Release formulation of Nimesulide (Willgo^®^) in ‘real world clinical practice’. For spontaneous reporting purpose the Suspected ADR reporting form provided by the CDSCO, Directorate General of Health Services, Government of India was used.

**Results:**

A total of 55 suspected ADR reporting forms were received. Maximum reporting was made from the Punjab state (16). Mean time taken for the assessment using WHO-UMC criteria was 7.17± 0.51 minutes and using Naranjo probability scale was 14.6 ± 3.13 minutes. In 47% cases a disagreement in causality assessment was found (k= 0.293).

**Conclusion:**

Consistent with the findings in the previous study (ies) a disagreement was found between the WHO-UMC Scale and the Naranjo scale. Among the two, the WHO-UMC scale is simple and less time consuming.

**096**

**Some interesting and unusual adverse drug reactions reported at Civil Hospital Ahmedabad, a tertiary care teaching hospital**

Prajapati SP, Patel PP, Desai CK, Desai MK, Dikshit RK

**B.J. Medical College and Civil Hospital, Ahmedabad 380016, India.**

**Introduction:**

As a part of continuous ADR Monitoring Program being carried out at the Department of Pharmacology, B. J. Medical College Ahmedabad, spontaneous reports of ADRs were collected from prescribers at associated Civil Hospital and the private practitioners from the city and recorded in Adverse Drug Reaction Monitoring Form (CDSCO). Some interesting and unusual ADRs found are reported here.

**Methods:**

Spontaneous reports received from June 2005 to May 2008 were analysed. The interesting and unusual ADRs were analysed on the basis of demographic criteria, clinical presentation, outcome and severity of ADRs.

**Results:**

A total number of 1139 ADRs were reported during the study period. Out of these, 45 interesting cases were observed in 43 patients. These include 2 cases of amenorrhea due to amisulipride and antiretroviral therapy (ART), anuria due to enalapril and rifampicin, IRIS (Immune Reconstitution Inflammatory Syndrome) due to ART, ciprofloxacin induced hypoglycaemia, atorvastatin induced pancreatitis, central serous retinopathy due to prednisolone and S.J.Syndrome due to multivitamins. Toxic Epidermal Necrolysis (TEN) (1), S.J.Syndrome (5), dermatographia (1) and angioedema (3) were observed due to drugs of unknown identity. Unintentional rechallenge with the causal drug was observed in 12 patients causing 14 ADRs.

**Conclusions:**

Interesting and unusual ADRs are usually reported spontaneously by the prescribers. It is however important to document these cases as they help create awareness among prescribers. These are important signal generating mechanisms in a pharmacovigilance program.

**097**

**Utilization of antihypertensives on the basis of demographic characteristics in a tertiary care hospital**

Datta S^1^, Udupa AL^2^, Kumar V^2^

**^1^Sikkim Manipal Institute of Medical Sciences, Gangtok, Sikkim, India; ^2^Kasturba Medical College, Manipal, Karnataka, India.**

**Introduction:**

Prevalence of hypertension cuts across age and gender barriers. Nevertheless, hypertension afflicts certain demographic groups more so than the others. In most populations, the risks of cardiovascular disease rise steeply with increasing age and at most ages is greater in men than in women. This study attempts to analyze variations in antihypertensive use on the basis of age and gender characteristics.

**Methods:**

A total of 303 prescriptions, having antihypertensives as a component were noted down at the Kasturba Medical College Hospital Pharmacy. Detailed patient information, pertaining to age and sex, was obtained from the Medical Records Department. Antihypertensives prescribed were analyzed in males and females, as well as in three age groups.

**Results:**

Amongst males and females, the calcium channel blockers were the most common group of drugs prescribed (74% and 64.9% respectively).ACE-inhibitors were prescribed in 31.7% males and 25.4% females respectively. Beta-blocker use was almost similar in males and females (29.1% and 30.7% respectively).ACE-inhibitors were the most commonly used drugs in the <17 years age group (60%). Calcium channel blockers were prescribed the most in the 18-65 and >65 years age group (72.8% and 70.7% respectively). Beta-blockers were prescribed more in the 18-65 age group (34.1%) than the >65 years age group (23.5%).Diuretics were prescribed more in the >65 years age group (35.9%) than 18-65 years age group (22.6%).

**Conclusion:**

Gender did not have a bearing on the selection of an antihypertensive agent. A distinctive pattern of use was observed for ACE-inhibitors, Beta-blockers and diuretics in the three age groups.

**098**

**Evaluation of adverse drug reaction reporting system in a tertiary care hospital, Greater Noida**

Sen S, Sinha S, Kamal CM, Bhardwaj VK

**Hindustan Institute of Medical/Dental Sciences and Research, Greater Noida, U.P., India**

**Introduction:**

Adverse drug reaction (ADRs) surveillance programme is necessary to detect, evaluate and develop mechanisms to prevent ADRs and their associated morbidity and mortality.

**Aim**

The study was aimed to design an efficient adverse drug reporting system and to evaluate its functioning in the hospital setup.

**Method:**

This was a prospective, spontaneous reporting study conducted over a period of one year. Adverse drug reaction data collected from all OPDs and IPDs of Sharda Hospital and HIDS Hospital, Greater Noida as per the proforma of the CDSCO, Govt. of India. The physicians and health workers notified the suspected ADRs and were assessed by pharmacologists through Naranjo’s causality assessment. Each ADR was assessed in relation to disease and drug therapy.

**Result:**

A total 89 suspected ADRs were reported and evaluated. Large number of ADRs were predictable and probable preventable. Suspected ADRs reported in 38.49% were mild, 52.81% moderate and 8.7 % severe. ADRs were mainly referable to CNS 7.8%, GIT 17% and cutaneous reactions 56%. The major category of drugs involved were NSAIDs and opioids (27%), floroquinolones (13%), beta lactam (11%), antiepileptics (4%), antitubercular (6%), antihypertensives (8%), anthelminthics (4%), cytotoxic medicines (8%) and others (19%).

**Conclusion:**

The reporting of suspected ADRs by physicians was implemented to promote rational and safe use of medicines. It serve as data base which can be utilized for predicting untoward reactions leading to improved patient care.This programme educated the health care professionals and patients about untoward drug effects and enhanced their level of awareness regarding ADRs.

**099**

**Current trend of utilization of evidence based medications among acs patients: A population based cross-sectional study**

^1^Pareek S, ^1^Balaraman R, ^2^Shah B, ^2^Parikh K

**^1^M.S.University Baroda, India, ^2^The Heartcare Clinic, Ahemedabad, India.**

**Objective:**

Our objective was to assess the current trend of utilization of key evidence-based drug therapies: Antiplatelet agents, ACEinhibitors/ARBs, BBs, and statins) for ACS patients and also to compare use of these therapies among diabetics and non diabetic patients population.

**Methods:**

This study was conducted at S.A.L. Hospital and Medical Research Institute, Ahmedabad, from Aug 2007 to Jan 2008, on370 Patients presented with NSTE-ACS.

**Result:**

Among all 30.5% patients were diabetic. Aspirin, ACEI/ ARBs, BBs, and statins were prescribed in95.4%, 97.6%, 83.0% and 91.9% of total patients. Among non-diabetic patients, 95.3%, 97.3%, 82.1% and 93.8% and among diabetic patients 95.6%, 98.2, 85.0% and 87.6% patients were prescribed aspirin, ACEI/ARBs, BBs, and statins respectively, on discharge. Difference in percentage of patients receiving various evidence-based drug therapies was not statistically significant among diabetic and non-diabetic patient populations (*P*>0.05); except for statins (*P*= 0.0454). Further ACEI and ARBs were evaluated separately for their use among patients with/with out diabetes. ARBs were more preferred among diabetic patients as compared to non-diabetic patients(12.4% vs. 6.6%); where asACEIs were more preffered among non-diabetics as compared to diabetics (79.4% vs. 73.5%). However, in either case difference in percentage of patients was not statistically significant (*P*<0.05).

**Conclusion:**

Overall use of evidence-based medications was consistent with published guidelines and recommendations by ACC/AHA for ACS patients. Non-diabetic patients were prescribed statins therapy more as compared to diabetics. Though overall utilization of ACEI was higher, considering its cardio- and renoprotective actions among diabetics, there is a potential room to improve its prescribing among diabetics.

**100**

**Prescribing patterns of antihypertensive drugs in a south Indian tertiary care hospital: A retrospective, cross sectional study in the in-patient wards**

Pai PG, Sanji N

**Kasturba Medical College, Manipal University, Mangalore, India.**

**Introduction:**

The choice of drug for the treatment of hypertension changes at short intervals. Drug utilization studies conducted at regular intervals help to guide the physician in prescribing drugs rationally. The present study was done to analyze the prescribing patterns of antihypertensive drugs in a South Indian tertiary care hospital.

**Methods:**

A retrospective, cross sectional analysis of prescriptions of antihypertensive cases admitted in Medicine in-patient wards of Kasturba Medical College, Hospital, Attavar, Mangalore during the period of January 2007 to July 2007 was conducted. All the prescription files with diagnosis of essential hypertension (ICD-9CM: 401-405, WHO international code: A 26) were analyzed. Prescriptions for hypertension with other co-morbid conditions were also included. Frequency and proportions of utilization of antihypertensive medications were charted and figured.

**Results:**

During the study period, thee were 200 prescriptions for essential hypertension. The most frequently prescribed antihypertensive medications were: Calcium channel blockers (49%) followed by diuretics (43.5%), angiotensin converting enzyme inhibitors (29.5%) beta blockers (29%)and angiotensin receptor blockers (21%). 51% of patients were on multiple drug therapy, the most favored fixed drug combination being diuretics with angiotensin receptor blockers (25.4%). Among the hypertensive cases with co-existing Diabetes mellitus type II, the most prescribed class of drugs was diuretics (43.8%) followed by angiotensin converting enzyme inhibitors (40.4%).

**Conclusion:**

The present study represents the current prescribing trend for antihypertensive agents. It implies that Calcium channel blockers are the leading group of antihypertensive agents followed by diuretics.

